# The Association Of Race With Outcomes Among Parturients Undergoing Cesarean Section With Perioperative Epidural Catheter Placement: A Nationwide Analysis

**DOI:** 10.7759/cureus.6652

**Published:** 2020-01-14

**Authors:** Ya-Ching Hsieh, Harsh R Shah, Pradeep Balasubramaniam

**Affiliations:** 1 Anesthesiology, The Icahn School of Medicine at Mount Sinai, New York, USA; 2 Anesthesiology, University of Connecticut School of Medicine, Farmington, USA; 3 Anesthesiology, Lincoln Medical Center, Bronx, USA

**Keywords:** cesarean, obstetrics, outcomes, racial disparity, nationwide inpatient sample, epidural anesthesia, population based research, nis, race, ethnicity

## Abstract

Background: In obstetrical health care, disparities have been documented in different aspects of maternal care and outcomes. Prior epidemiological studies have shown that labor analgesia is underused in African-American and Hispanic groups, which means there may be inadequate labor pain control in these groups. Differences in usage have been attributed primarily to insurance, educational levels and perceptional influences such as fear of paralysis and chronic low back pain. In cesarean section deliveries, race and ethnicity affect the choice of anesthesia considered. How race and ethnicity affect maternal outcomes in cesarean sections with epidural placements generally has been unexplored. Disparities in health care utilization are shown to contribute to the disparities in health outcomes.

Methods: This is a retrospective analysis using data from the Agency for Healthcare Research and Quality Healthcare Cost and Utilization Project (AHRQ-HCUP), the National Inpatient Sample (NIS) database from January 2003 to December 2013, which is a 20% stratified sample of the nonfederal hospitals in the United States. Women undergoing cesarean section (International Classification of Diseases, 9th Revision, Clinical Modification (ICD-9-CM) procedure codes 74.0, 74.1, 74.2, 74.4, 74.99) with perioperative epidural catheter placement (ICD-9-CM procedure codes 3.90, 3.91) were included for analysis.

Results: The final cohort used for analysis included 87,076 patients. There were significant differences in the distribution of patient characteristics across the race groups. The majority of health care coverage for Caucasians and Asians was private insurance, while for African-American, Hispanic and Native American was Medicare and Medicaid. Almost all the examined comorbid conditions were statistically significant and highest in the African-American group, including hypertension, obesity, diabetes, and renal failure, except for congestive heart failure that was highest in the Asian group. Cesarean sections took place mostly in an urban teaching hospital across all groups. Discharge to home was the predominant destination after recovery. The mean cost of hospitalization was 14,604 dollars per stay and the mean length of stay was 3.7 days. In our cohort, the adverse event rate was very small. Our findings indicate racial differences in comorbidities which occurred more often in minorities. Adverse maternal outcomes of hematoma, blood transfusion, cardiac arrest, and ventricular fibrillation occurred more frequently in minority groups undergoing cesarean sections with epidural catheter placements throughout the period of 2003-2013.

Conclusion: From using the NIS database, our findings indicate racial differences in comorbidities which occurred more often in minorities. Adverse maternal outcomes of hematoma, blood transfusion, cardiac arrest, and ventricular fibrillation occurred more frequently in minority groups undergoing cesarean sections with epidural catheter placements throughout the period of 2003-2013. Further population studies are warranted to determine the biological or perception etiologies that are contributing to these disparities.

## Introduction

Racial and ethnic disparities exist in many areas of health and health care. Examination on the local and national levels of these health disparities is central to determining the extent of health inequities affecting minorities. The focus on race is an important aspect in improving healthcare outcomes since the populations that are generally being underserved in the United States are African-Americans, Hispanics, Asians, and Native Americans. Though the common belief that health disparities occur because of lack of insurance and access to care, this still remains even after these factors are removed. Racial and ethnic minorities are growing populations in the United States, therefore, there is a need for adequately addressing their poor health status and these persistent disparities at a time of rapidly racial and ethnic diversification.

In obstetrical health care, disparities have been documented in different aspects of maternal care and outcomes [1-3]. Prior epidemiological studies have shown that labor analgesia is underused in African-American and Hispanic groups, which means there may be inadequate labor pain control in these groups [4-5]. Differences in usage have been attributed primarily to insurance, educational levels and perceptional influences such as fear of paralysis and chronic low back pain [6-7].

When normal delivery is complicated by factors either maternal or fetal, a cesarean section is warranted to ensure maternal and fetal safety. For cesarean sections, there has been a shift in the practice of anesthesia from general to neuraxial as the preferred modality of analgesia [8]. In cesarean section deliveries, race and ethnicity affect the choice of anesthesia considered. How race and ethnicity affect maternal outcomes in cesarean sections with epidural placements generally has been unexplored. Disparities in health care utilization are shown to contribute to the disparities in health outcomes [9]. Examining race as a factor affecting outcomes of this common obstetrical procedure done under epidural analgesia will contribute to the understanding of maternal morbidity and mortality.

## Materials and methods

This is a retrospective analysis using data from the Agency for Healthcare Research and Quality Healthcare Cost and Utilization Project (AHRQ-HCUP), the National Inpatient Sample (NIS) database from January 2003 to December 2013, which is a 20% stratified sample of the nonfederal hospitals in the United States, representing more than 95% of the national population. Each hospitalization is treated as an individual entry in the database and is coded with one principal diagnosis, up to 24 secondary diagnoses, and 15 procedural diagnoses associated with that stay. Detailed information on NIS is available at http://www.hcup-us.ahrq.gov/db/nation/nis/nisdde.jsp.

We used the 9th revision of the International Classification of Diseases, Clinical Modification (ICD-9-CM) codes to identify women undergoing cesarean section (ICD-9-CM procedure codes 74.0, 74.1, 74.2, 74.4, 74.99) with perioperative epidural catheter placement (ICD-9-CM procedure codes 3.90, 3.91). Age <18 years and admissions with missing data for age, sex, and race were excluded.

Our proposed outcomes were total cost, all causes of in-hospital mortality, discharge disposition, and complications related to admissions. Complications included in analysis were as follows: hematoma (998.12), surgical site infection (998.59), postpartum hemorrhage (666.14, 666.24, 661.24, 666.34), blood transfusion (99.00, 99.09), severe anesthesia complications (668.0, 668.1, 668.2, 668.8, 668.9), amniotic fluid embolism (673.1), disseminated intravascular coagulation (286.6, 286.9, 666.3), pulmonary edema (428.1, 518.4) and cardiac arrest/ventricular fibrillation (427.41, 427.42, 427.5).

Weighted univariate analysis was used to analyze and summarize the baseline and demographic characteristics of the parturient cohort. The chi-square test and paired student t-test were used to examine the association between independent variables and outcomes of interest. All statistical tests used were two-sided, and p<0.05 was deemed statistically significant. No statistical power calculation was conducted prior to the study.

## Results

In this study cohort, 91,053 women underwent a cesarean section with perioperative epidural catheter placement. The final cohort used for analysis included 87,076, excluding those who had missing data for at least one of the covariates. Table 1 shows the clinical characteristics of the study population in the different racial groups. The overall distribution of race was 63% Caucasian, 13% African American, 20% Hispanic, 3% Asian and 1% Native American. There were differences in the distribution of demographic characteristics across the race groups. Between the groups, the Asian mothers had the highest percentage of age above 35 (23%). The majority of health care coverage for Caucasians (62%) and Asians (66%) was private insurance, while for African-American (67%), Hispanic (69%) and Native American (56%) was Medicare and Medicaid. Almost all the examined comorbid conditions were statistically significant and highest in the African-American group, including hypertension (8%), obesity (9%), diabetes (2.48%), and renal failure (0.12%), except for congestive heart failure that was highest in the Asian (0.2%) group. The cesarean sections took place mostly in an urban teaching hospital across all groups, with frequencies ranging from 43% in Caucasians to 82% in African-Americans. Discharge to home was the predominant destination after recovery, occurring in at least 94% of subjects in all racial groups. The mean cost of hospitalization was 14,604 dollars per stay and the mean length of stay was 3.7 days.

**Table 1:** Univariate analysis of the epidemiological characteristics of women who have undergone a cesarean section with epidural anesthesia Percentages in brackets are column % that indicate the direct comparison of racial disparity among women who have undergone a cesarean section with epidural anesthesia. p-values were calculated based on racial groups: Caucasian, African American, Hispanic, Asian, and Native American.

	Total (n=87,076)	Caucasian (n=55,098)	African American (n=11,649)	Hispanic (n=16,977)	Asian (n=2,515)	Native American (n=837)	p-values	
SOCIODEMOGRAPHICS									
Age group (year)							<0.0001	
18-34	71,109	46,066 (85)	9,308 (84)	13,133 (80)	1,911 (77)	691 (85)		
35-49	13,565	7,822 (15)	1,824 (16)	3,230 (20)	567 (23)	122 (15)		
50-64	15	<10 (0)	<10 (0)	0 (0)	<10 (0)	0 (0)		
Insurance							<0.0001	
Medicare/ Medicaid	38,722	18,125 (33)	7,797 (67)	11,630 (69)	699 (28)	471 (56)		
Private	42,197	34,152 (62)	2,646 (23)	3,427 (20)	1,662 (66)	311 (37)		
Other	5,986	2,661 (5)	1,196 (10)	1,921 (11)	154 (6)	55 (7)		
Location/Teaching Status							<0.0001	
Rural	15,347	12,802 (23)	340 (3)	1,200 (7)	683 (27)	322 (38)		
Urban non-teaching	23,629	18,795 (34)	1,731 (15)	2,471 (15)	551 (22)	80 (10)		
Urban teaching	48,100	23,501 (43)	9,577 (82)	13,307 (78)	1,280 (51)	435 (52)		
Comorbidities								
Hypertension	3,264	1,719 (3)	894 (8)	564 (3)	51 (2)	36 (4)	<0.0001	
Obesity	5,050	3,002 (5)	1,077 (9)	874 (5)	35 (1)	62 (7)	<0.0001	
Congestive heart failure	41	27 (0.1)	<10 (0.1)	0 (0)	<10 (0.2)	0 (0)	0.0002	
Diabetes	1,358	707 (1.28)	288 (2.48)	334 (1.97)	14 (0.56)	15 (1.79)	<0.0001	
Renal failure	48	24 (0.04)	14 (0.12)	10 (0.06)	0 (0)	0 (0)	0.014	
Coagulopathy	1,087	686 (1.25)	156 (1.34)	210 (1.23)	30 (1.21)	<10 (0.61)	0.45	
ADVERSE OUTCOMES									
Hematoma	110	46 (0.08)	13 (0.11)	47 (0.28)	<10 (0.17)	0 (0)	<0.0001	
Surgical site infection	34	29 (0.05)	0 (0)	<10 (0.03)	0 (0)	0 (0)	0.051	
Postpartum hemorrhage	<10	<10 (0.01)	0 (0)	0 (0)	0 (0)	0 (0)	0.54	
Blood transfusion	15	0 (0)	5 (0.04)	10 (0.06)	0 (0)	0 (0)	<0.0001	
Severe anesthesia complications	851	560 (1.02)	71 (0.61)	171 (1.01)	19 (0.76)	30 (3.58)	<0.0001	
Amniotic fluid embolism	13	<10 (0.01)	0 (0)	<10 (0.03)	0 (0)	0 (0)	0.41	
Disseminated intravascular coagulation	315	194 (0.35)	34 (0.29)	73 (0.43)	14 (0.57)	0 (0)	0.04	
Pulmonary edema	60	35 (0.06)	<10 (0.04)	20 (0.12)	0 (0)	0 (0)	0.047	
Cardiac arrest/ventricular fibrillation	9	<10 (0.01)	<10 (0.05)	0 (0)	0 (0)	0 (0)		
In-hospital Mortality	10	<10 (0.01)	<10 (0.05)	0 (0)	0 (0)	0 (0)	0.004	
Discharge Destination							<0.0001	
Home	83,302	52,246 (95)	11,446 (98)	16,436 (97)	2,373 (94)	801 (96)		
Short term/ Intermediate care	3,769	2,852 (5)	203 (2)	537 (3)	141 (6)	36 (4)		
Total Charge	14,604	12,798	18,559	17,725	13,683	15,643	<0.0001	
Length of Stay (day)	3.7	3.6	4.1	3.9	3.7	3.6	<0.0001	

Rates of adverse events were very small in the overall cohort, with severe anesthesia complications occurring most frequently with the highest percentage in Native Americans (3.58%), followed by disseminated intravascular coagulation with the highest percentage in Asian (0.57%), and hematoma with the highest percentage in Hispanics (0.28%). Infrequent occurrences of adverse events included pulmonary edema (60), surgical site infection (34), blood transfusion (15), amniotic fluid embolism (13), cardiac arrest/ventricular fibrillation (9) and postpartum hemorrhage (5). Due to the small rates of adverse events, with multivariate analysis the models were unstable and therefore only unadjusted results are presented.

## Discussion

Using clinical data from a national database, we analyzed over 87,076 women who had cesarean section deliveries with perioperative epidural catheter placements from hospitals across the United States over the period of 2003 to 2013, and our overall results were indicative of certain racial differences in baseline maternal demographics, e.g. with the majority of African-Americans, Hispanics and Native Americans having Medicare or Medicaid for health insurance while private insurance for the majority of Caucasians and Asians.

The general demographic findings of demographic characteristics are similar to other prevalence studies with rates of complications being very small across the different population groups; however, minority group mothers face more complications in labor and delivery [2,10]. In 2007, Glance et al. found that, in New York State, African-American mothers were at increased odds of systemic anesthesia-related complications during Cesarean deliveries [4]. In our study, Native Americans had the highest percentage of severe anesthesia complications instead. However, the growing numbers of studies suggest a trend where the African-American and Hispanics minority groups have inadequate health care coverage which can, in turn, result in poorer outcomes in these respective groups [11-12]. The difference in minority groups with an increased number of anesthesia complications may have resulted from the differing sets of anesthesia-related severe complications used for analysis as compared to other studies.

There are inadequate labor analgesia and a greater number of epidural failures based on a parturient’s race [13]. While this phenomenon was not part of the main goal of our study, we did not find that race was a factor that had affected maternal outcomes in surgical site infection, postpartum hemorrhage or amniotic fluid embolism. We did note that African-Americans were burdened with more comorbidities at baseline, which could account for poorer outcomes found in the other studies.

This study is a large population cohort study to examine maternal complications in a common surgical procedure and anesthesia modality. The findings can have important public health and clinical relevance in influencing health care policies. Epidural catheter placements have become a widely used mode of analgesia during the period of labor and delivery. General findings of higher maternal mortality and morbidity among African-American and Hispanics as compared to Caucasians need to be studied, especially how race plays a role in health care delivery and their outcomes [11-14]. Though morbidity and mortality from anesthesia during the cesarean section have decreased in recent times, African-American women still suffer from a higher incidence of maternal death [13], and in our study, a slightly higher percentage of cardiac arrest and ventricular fibrillation. The Institute of Medicine had examined racial disparities in healthcare in the United States [15]. They reported that these healthcare disparities were rooted in historic and contemporary inequities in the healthcare systems and financing, care providers and patient interactions, attitudes, beliefs, and perceptions. In our study, we posit the differences could be attributable to African-Americans having higher numbers of comorbid conditions going into pregnancy, which would likely have an impact on the duration of gestation and the outcomes. There is limited research into the etiologies for the disparities, and it remains unknown whether biases at the patient level, healthcare systems-level or health care provider level are most significantly driving the disparity.

There were a number of limitations to consider for this study. With NIS data, an entry is equivalent to a single hospitalization. In obstetrical practice, there is a debate on the practice of vaginal deliveries after prior cesarean section delivery. A very likely scenario would be a parturient with a history of prior cesarean section undergoing multiple cesarean section deliveries. The consequent hospitalization data entries would be considered separate entries for the particular admission, which may or may not distort the outcomes depending on how often it occurs.

In the NIS data it cannot be determined when an epidural catheter was placed, whether placed as a “walking epidural” to ease labor pains for vaginal delivery and due to a failed attempt converted to cesarean section, or it was placed in the operating room specifically planned for the procedure. This could have some bearing on the adverse events.

By assessing adverse outcomes using the ICD-9 coding system as recorded in the NIS database, it is uncertain if a particular diagnosis was a prior diagnosis in the patient’s medical history or if the diagnosis occurred during the specific hospitalization. This can distort the true rate of the complications in the analysis.

Despite the cohort comprising of a large number of women, there were significantly lower subpopulations of Asians and Native Americans, which, coupled with missing data, resulted in insignificant odds ratios and extremely wide confidence intervals in regression analyses. Further population studies could focus more on comorbid conditions of the parturients during gestation and its impact on maternal mortality and morbidity. 

A major strength of the study was that findings were nationally representative for the United States. NIS data is the largest inpatient database, and our study has good statistical power. The limitation is that the data from clinical registries are obtained retrospectively by chart abstractions based on the discharge diagnosis codes, billing codes, etc. and hence susceptible to coding errors.

## Conclusions

From using the NIS database, our findings indicate racial differences in comorbidities which occurred more often in minorities. Adverse maternal outcomes of hematoma, blood transfusion, cardiac arrest, and ventricular fibrillation occurred more frequently in minority groups undergoing cesarean sections with epidural catheter placements throughout the period of 2003-2013. Further population studies are warranted to determine the biological or perception etiologies that are contributing to these disparities.
